# Supplementary material for: A neural circuit for gamma-band coherence across the retinotopic map in mouse visual cortex
Source: eLife. 2018 Feb 26;7:e28569. doi: 10.7554/eLife.28569 (PMC5826269; doi:10.7554/eLife.28569)
Supplement: Transparent reporting form [file elife-28569-transrepform.docx]

***eLife’s* transparent reporting form**

We encourage authors to provide detailed information *within their submission* to facilitate the interpretation and replication of experiments. If you have any questions, please contact us: [editorial@elifesciences.org](mailto:editorial@elifesciences.org).

**Sample-size estimation**

- You should state whether an appropriate sample size was computed when the study was being designed
- You should state the statistical method of sample size computation and any required assumptions
- If no explicit power analysis was used, you should describe how you decided what sample (replicate) size (number) to use

Please outline where this information can be found within the submission (e.g., page numbers or figure legends), or explain why this information doesn’t apply to your submission:

Prior experience and preliminary experiments demonstrated large effect sizes for all experiments, as such, estimations of sample sizes necessary to achieve statistical significance were made instead of formal power analysis.

Extended information:

**Fig 1C:** Based on prior experience (see Adesnik et al 2012), and a large consistent effect-size, we estimated that we needed at least 6 samples for this experiment to overcome variability and achieve a statistically significant result. n=8.

**Fig 1J:** Based on the large effect size seen during preliminary experiments, we estimated that we needed at least 6 samples for each condition in the experiment to overcome variability and achieve a statistically significant result. n=6 ‘close’ condition, n=7 ‘far’ condition, n=11 ‘cut’ condition.

**Fig 2C:** Based on prior experience (see Adesnik et al 2012), we estimated that we needed at least 8 samples for this experiment to overcome variability and achieve a statistically significant result. n=10 SOM, n=11 PV.

**Fig 2E:** Based on prior experience (see Adesnik et al 2012), and a large consistent effect-size, we estimated that we needed at least 6 samples in each condition for this experiment to overcome variability and achieve a statistically significant result. n=12 SOM, n=8 PV, n=11 PC.

**Fig 3C:** Based on prior experience (see Adesnik et al 2010, 12), and a large consistent effect-size, we estimated that we needed at least 6 samples in each condition for this experiment to overcome variability and achieve a statistically significant result. n=24 IPSC, n=9 EPSC.

**Fig 4D:** Based on the large effect size seen during preliminary experiments, we estimated that we needed at least 6 samples in the experiment to overcome variability and achieve a statistically significant result. n=8.

**Replicates**

- You should report how often each experiment was performed
- You should include a definition of biological versus technical replication
- The data obtained should be provided and sufficient information should be provided to indicate the number of independent biological and/or technical replicates
- If you encountered any outliers, you should describe how these were handled
- Criteria for exclusion/inclusion of data should be clearly stated
- High-throughput sequence data should be uploaded before submission, with a private link for reviewers provided (these are available from both GEO and ArrayExpress)

Please outline where this information can be found within the submission (e.g., page numbers or figure legends), or explain why this information doesn’t apply to your submission:

For all experiments, slices were screened for expression by looking for fluorescence in the visual cortex and by measuring large oscillatory currents with a clear peak in the gamma range in response to ramping blue light stimuli. This information is found in the opening of the results section.

Sample sizes are included in the text and figure legends.

Extended sample size information:

**Fig 1C:** 8 cells from 8 slices 4 animals.

**Fig 1J:** Close condition: 6 pairs from 6 slices from 3 animals.

Far condition: 7 pairs from 6 slices from 3 animals.

Cut condition: 11 pairs from 7 slices from 6 animals.

**Fig 2C:** SOM spiking: 10 cells from 5 slices from 3 animals.

PV spiking: 11 cells from 5 slices from 3 animals.

**Fig 2E:** SOM cells: 12 cells from 5 slices from 3 animals.

PV cells: 8 cells from 3 slices from 3 animals.

Pyramidal cells: 11 cells from 4 slices from 3 animals.

**Fig 3C:** IPSC: 24 cells from 13 slices from 8 animals.

EPSC: 9 cells from 7 slices from 4 animals (subset of the cells used for IPSC recordings). EPSC recordings were included if the peak gamma power of the EPSCs was greater than 20pA^2/Hz and if clear oscillations could be observed in the IPSC recordings.

**Fig 4D:** 8 pairs from 4 slices from 3 animals.

Outliers were included.

**Statistical reporting**

- Statistical analysis methods should be described and justified
- Raw data should be presented in figures whenever informative to do so (typically when N per group is less than 10)
- For each experiment, you should identify the statistical tests used, exact values of N, definitions of center, methods of multiple test correction, and dispersion and precision measures (e.g., mean, median, SD, SEM, confidence intervals; and, for the major substantive results, a measure of effect size (e.g., Pearson's r, Cohen's d)
- Report exact p-values wherever possible alongside the summary statistics and 95% confidence intervals. These should be reported for all key questions and not only when the p-value is less than 0.05.

P-values were reported in the text as exact values if p>0.01, or as p<10^-x if rounding up resulted in x<=2.

Definitions of center are described in text and figure legends. Definition of variance in text (y in ‘x±y’) are SEM. Definitions of variance/errorbars in figures are described in figure legend.

Extended statistics:

**Fig 1C:**

Kruskal-Wallis ANOVA on mean values of peak gamma power in each cell at each stimulus size:

Source	SS	df	MS	Chi-sq	Prob>Chi-sq

------------------------------------------------------

Columns	6104.5	5	1220.9	31.29	**8.21699e-06**

Error	3065.5	42	72.99

Total	9170	47

**Fig 1J:**

Wilcoxon rank sum test between mean values of peak gamma power from ‘close’ and ‘cut’ conditions:

**p = 3.9960e-04**; ranksum = 45;

Wilcoxon rank sum test between mean values of peak gamma power from ‘far’ and ‘cut’ conditions:

**p = 3.4965e-04**; ranksum = 46;

Kruskal-Wallis ANOVA on mean values of peak gamma power from all conditions:

Source	SS	df	MS	Chi-sq	Prob>Chi-sq

------------------------------------------------------

Groups	686.421	2	343.21	16.28	**0.0003**

Error	199.079	19	10.478

Total	885.5	21

(continued onto next page)

Please outline where this information can be found within the submission (e.g., page numbers or figure legends), or explain why this information doesn’t apply to your submission:

(continued)

**Fig 2C:**

Wilcoxon rank sum test between mean values of spike rates from PV and SOM cells:

**p = 0.0021**; zval = -3.0789; ranksum = 78;

**Fig 2E:**

Wilcoxon rank sum test between PV and SOM cell E/I ratios:

**p = 2.7784e-04**; ranksum = 31;

Kruskal-Wallis ANOVA on mean values of E/I ratio among PV, SOM, and Pyramidal cells:

Source	SS	df	MS	Chi-sq	Prob>Chi-sq

------------------------------------------------------

Groups	1721.54	2	860.768	23.75	**6.97861e-06**

Error	308.46	26	11.864

Total	2030	28

**Fig 3C:**

Wilcoxon signed rank test between peak gamma power in IPSCs with light on (SOM suppression) and light off (control):

**p= 8.2842e-06**; zval: 4.4577; signedrank: 351

Wilcoxon signed rank test between peak gamma power in IPSCs with light on (SOM suppression) and light off (control):

**p = 0.0039**; signedrank: 45

**Fig 4D:**

Wilcoxon signed rank test between PPC (measure of spike entrainment to oscillation, see methods) with light on (SOM suppression) and light off (control):

**p = 0.0039**; signedrank: 45

(For large datasets, or papers with a very large number of statistical tests, you may upload a single table file with tests, Ns, etc., with reference to page numbers in the manuscript.)

**Additional data files (“source data”)**

- We encourage you to upload relevant additional data files, such as numerical data that are represented as a graph in a figure, or as a summary table
- Where provided, these should be in the most useful format, and they can be uploaded as “Source data” files linked to a main figure or table
- Include model definition files including the full list of parameters used
- Include code used for data analysis (e.g., R, MatLab)
- Avoid stating that data files are “available upon request”

Please indicate the figures or tables for which source data files have been provided:
